# Maternal diet, nutritional status and infant birth weight in Malaysia: a scoping review

**DOI:** 10.1186/s12884-022-04616-z

**Published:** 2022-04-06

**Authors:** Hamid Jan Jan Mohamed, See Ling Loy, Amal K. Mitra, Satvinder Kaur, Ai Ni Teoh, Siti Hamizah Abd Rahman, Maria Sofia Amarra

**Affiliations:** 1 Nutrition and Dietetics Programme, School of Health Sciences, Universiti Sains Malaysia, Health Campus, 16150 Kubang Kerian, Kelantan Malaysia; 2 Department of Reproductive Medicine, KK Women’s and Children’s Hospital, Singapore, 229899 Singapore; 3 Duke-NUS Medical School, Singapore, 169857 Singapore; 4 Department of Epidemiology and Biostatistics, School of Public Health, College of Health Sciences, Jackson State University, Jackson, MS 39213 USA; 5 Faculty of Applied Sciences, UCSI University, Wilayah Persekutuan Kuala Lumpur, 56000 Kuala Lumpur, Malaysia; 6 Department of Food Science and Nutrition, University of the Philippines Diliman, 1101 Quezon City, Philippines; 7 School of Nutrition, Philippine Women’s University, Taft Avenue, Manila, 1004 Philippines

**Keywords:** Dietary intake, Body mass index, Gestational weight gain, Gestational diabetes, Gestational hypertension, Low birth weight, Macrosomia, Pregnancy

## Abstract

**Background:**

Women’s diet and nutritional status during pregnancy are important in influencing birth outcomes. We conducted a systematic scoping review of the best available evidence regarding dietary intake of Malaysian pregnant women, and the associations of maternal diet, anthropometry, and nutrition-related co-morbidities with the infant’s birth weight (IBW). The study objectives were to examine: (1) the adequacy of micronutrient intake among pregnant women; and (2) the association of maternal factors (anthropometry, diet, plasma glucose and blood pressure) during pregnancy with IBW.

**Methods:**

Eleven search engines such as Proquest, EbscoHost, Scopus, Cochrane Library, Science Direct, Wiley Online Library, PubMed, Google Scholar, MyJournal, BookSC and Inter Library Loan with Medical Library Group were extensively searched to identify the primary articles. Three reviewers independently screened the abstracts and full articles based on the inclusion and exclusion criteria. Extracted data included details about the population characteristics, study methods and key findings related to the review objectives. Seventeen studies published from 1972 to 2021 were included, following the PRISMA-ScR guideline.

**Results:**

Studies showed that maternal micronutrient intakes including calcium, iron, vitamin D, folic acid, and niacin fell short of the national recommendations. Increased maternal fruit intake was also associated with increased birth weight. Factors associated with fetal macrosomia included high pre-pregnancy body mass index (BMI), excess gestational weight gain (GWG) and high blood glucose levels. Low pre-pregnancy BMI, inadequate GWG, intake of confectioneries and condiments, and high blood pressure were associated with low birth weight.

**Conclusion:**

This review identified several factors such as the mother’s food habits, comorbidities, BMI and gestational weight gain as the determinants of low birth weight. This implies that emphasis should be given on maternal health and nutrition for the birth outcome.

## Introduction

The nutritional status of women prior to conception and during pregnancy is important for fetal growth and development. The first 1000 days of life (i.e., the period of conception up to the first two postnatal years) emphasize the importance of optimal maternal nutritional status in determining early infant development and risk of nutrition-related chronic diseases in adulthood [1]. Maternal undernutrition and insufficient gestational weight gain (GWG) are key contributors to increased incidence of preterm birth (PTB), low birth weight (LBW) and poor fetal growth [2, 3]. On the other hand, maternal obesity, adiposity and excessive GWG are associated with several issues in child birth and subsequent health of the child including caesarean section delivery, late antepartum death, excessive fetal growth, macrosomia and childhood obesity [4–6]. There is no published national data on macrosomia in Malaysia. Maternal micronutrient deficiencies such as iron, folic acid, zinc, vitamin A and vitamin D are also found to impose adverse effects on pregnancy and infant outcomes.

In May 2012, the 65th World Health Assembly endorsed the Comprehensive Implementation Plan on Maternal, Infant and Young Child Nutrition (MIYCN). This plan specified a set of six global nutrition targets, including a 30% reduction of LBW. Based on a nationwide survey done among Malaysians, 1 out of 10 livebirth babies were born LBW (NHMS 2016). This is similar to neighboring countries Thailand (9.2%) but lower than Indonesia (20.2%) [7]. While there are several factors underlying the LBW occurrence, understanding maternal diet and nutritional-related factors during pregnancy serve as an important indicator to modifiable risks. Given that Malaysia is unique with its multiracial and multicultural practice in eating, it is important to recognize how maternal nutrition influences pregnancy outcomes in this country. This will facilitate effort to address the challenge of local maternal nutritional issue for ensuring optimal pregnancy outcomes and subsequently, infant health. Hence, addressing the challenge of maternal nutritional status is a major priority in the region ensuring optimal pregnancy outcomes and subsequently, infant health.

The scoping review was conducted to better understand the areas of priority research in maternal diet and nutritional status of the mother and the child in Malaysia. For those research needs, the scoping review was done for the case of Malaysia, because of the unavailability of data on maternal nutritional status with low birth weight in the country. The present review aims to examine the best available evidence regarding dietary intake of Malaysian pregnant women, and the association of diet, anthropometry, and existing co-morbidities during pregnancy with birth outcomes. The objectives are to evaluate: 1) The adequacy of selected dietary micronutrient intake among pregnant women; 2) The association of the following maternal nutritional factors on infant’s birth weight defined in terms of macrosomia and LBW: pre-pregnancy body mass index (BMI), and GWG; 3) Maternal food group intake; and 4) Selected co-morbidities during pregnancy with the infant’s birth weight, such as maternal high blood glucose and high blood pressure.

## Materials and methods

The scoping review framework by Arksey and O’Malley was used as a guide to conduct the review [8]. The Preferred Reporting Items for Systematic Reviews and Meta-Analysis extension for scoping reviews (PRISMA-ScR) guidelines (2018) was followed to report this study [9]. The protocol for this review has not been registered.

### Identifying relevant studies

The data search strategy comprised of primary studies, grey literature and annual reports published from 1972 to 2021. The following databases were used: Proquest, EbscoHost, Scopus, Cochrane Library, Science Direct, Wiley Online Library, PubMed, Google Scholar, MyJournal (an online system provided by Malaysia Citation Centre of Ministry of Education), BookSC and Inter Library Loan with Medical Library Group. Search terms used were: Malaysia, maternal nutrition, diet, pregnancy, birth outcome, birth weight, macrosomia, premature, low birth weight, intrauterine growth restriction, gestational diabetes, plasma glucose, blood pressure, obesity, gestational weight gain, anemia, folic acid deficiencies and iron deficiencies. The last search was performed on 25 August 2021. The search strategy is shown in Table 1.

**Table 1:** Search strategies and research threads for databases

Search Strategy	No. of Studies Available
Key words used: Malaysia, maternal nutrition, diet, pregnancy, birth outcome, birth weight, macrosomia, premature, low birth weight, intrauterine growth restriction, gestational diabetes, plasma glucose, blood pressure, obesity, gestational weight gain, anemia, folic acid deficiencies and iron deficiencies. Example 1: Google Scholar search using Malaysia AND maternal nutrition AND diet AND pregnancy AND birth outcome AND birth weight AND macrosomia AND premature AND low birth weight yielded 794 records. Example 2: PubMed search using Malaysia AND maternal nutrition AND diet AND pregnancy AND low birth weight yielded 12 records.	893 from the primary sources and 4 from additional databases.
Duplicate records removed	728 remained after duplicates removed
Total number of studies which did not meet the objectives of study were removed (*n* = 599)	129 remained
Studies (*n* = 99) removed with reasons	30 studies remained
Studies having no full text (*n* = 13) were excluded	17 studies remained for the final analysis

### Inclusion criteria

**Fig. 1:**
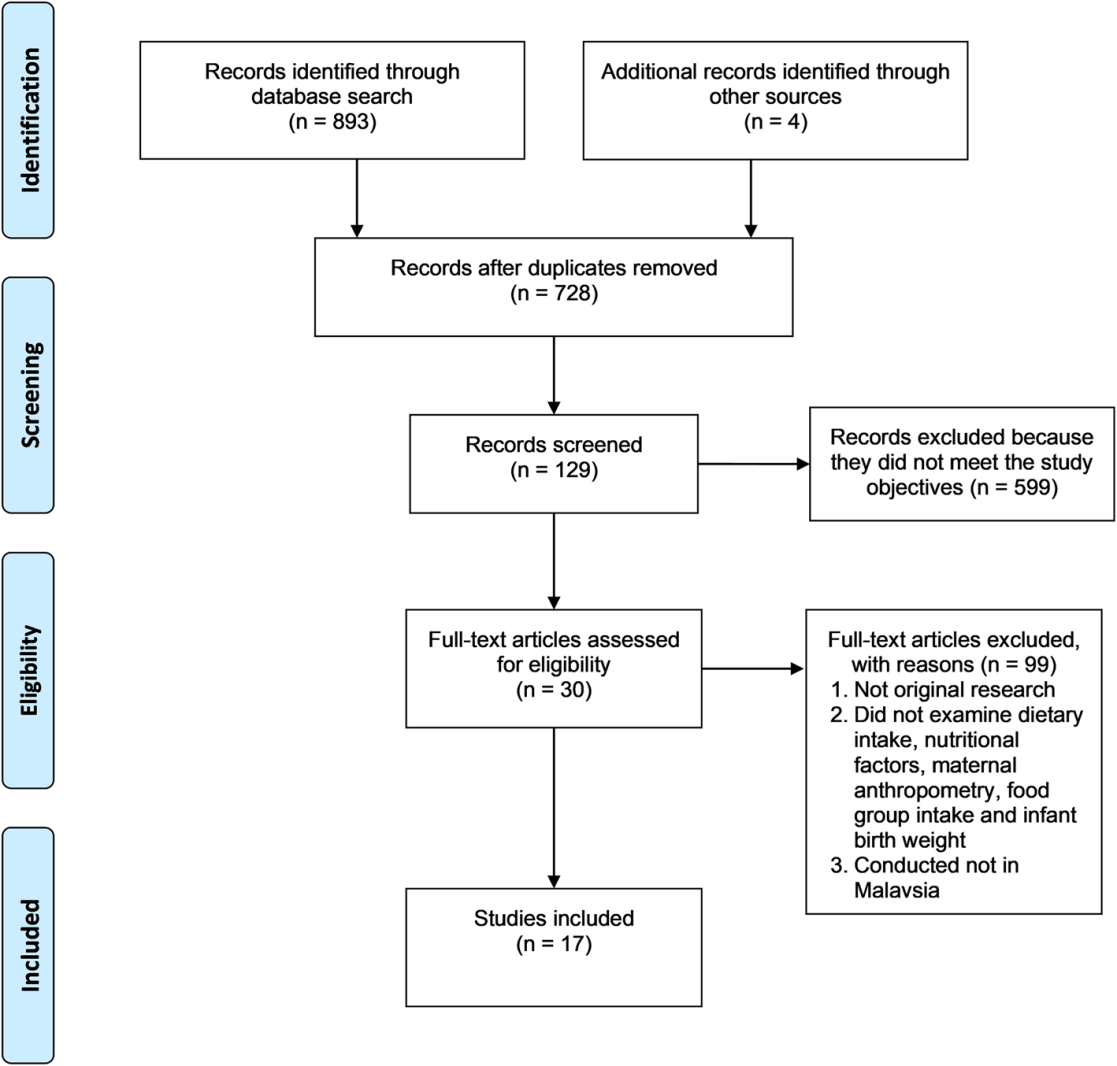
PRISMA Flowchart showing the results of search strategy and inclusion and exclusion of articles

The screening process was conducted in accordance with the scoping review framework by Arksey and O’Malley and recommendations made by Levac et al. [8, 10]. Three researchers (HJ, SK and SL) independently screened the titles and retrieved the abstracts based on the inclusion criteria and exclusion criteria as mentioned in Table 2. Selected abstracts were reviewed to assess their eligibility for full text review. Subsequently, the full text articles of eligible abstracts were retrieved and assessed independently by three reviewers to determine whether they answered the specific research questions for this review and fulfilled the inclusion criteria. Studies were included in the review if consensus was achieved by all three researchers. Figure 1 shows a schematic diagram for the selection of articles in this review.

**Table 2:** Inclusion and exclusion criteria

Inclusion Criteria	Exclusion Criteria
Quantitative studies	Review articles
Human studies	Animal studies
Scholarly paper	Study conducted outside Malaysia
Published between 1972 and 2021	
Native and English language

### Charting and summarizing the data

Data were extracted independently by three reviewers. To map the existing literature on the review objectives, findings were grouped by adequacy of dietary intake, nutritional factors and infant birth weight, maternal anthropometry and birth weight (pre-pregnancy BMI, GWG, Mid-upper arm circumference), food group intake and birth weight as well as nutrition-related co-morbidities in pregnancy and birth weight. General and specific information about the studies which included author(s), year of publication, study design, sample characteristics, sample size, exposure and outcomes that were relevant to the objectives of the review were charted in Table 2.

### Quality Appraisal

Studies were appraised by three researchers for quality [8–11]. Three researchers (HJ, SK, and SL) independently evaluated the quality of the 17 studies from a rating scale of 0 to 4 based on the following criteria: (a) Study design: studies employing cross-sectional, case–control, or cohort design = 1, otherwise = 0; (b) Sample size: large = 1, small = 0; (c) Use of validated questionnaires or standardized tools for data collection, such as measurement of dietary intake using Food Frequency Questionnaire (FFQ) = 1, not specific or otherwise = 0; and (d) Sampling method: random sampling or reduced bias = 1, non-random or convenience sampling or presence of bias = 0 [12]. An average value of the three scores was presented as the final score because there were no significant inter-observer variations in the assessment of the quality of the included articles. The scores were grouped as follows: 1 = poor; 2 = moderate; 3–4 = high quality.

## Results

Out of the 17 studies that were included in the review, 8 studies investigated infant birth weight while only 4 studies included macrosomia as the outcome variable. There were 4 studies that examined the adequacy of micronutrient intakes and 1 study investigated food group intake among pregnant women. Table 3 shows the characteristics of the studies that were included, sorted in the order of the publication date.

**Table 3:** Sample size, participant characteristics, study design, quality appraisal, and exposure and outcome variables

Study (year)	Sample size	Participant characteristics	Study design	Quality Appraisal (out of 4)	Exposure		Outcome		Results
					Method of data collection	Variables examined	Timing	Variable	
Boo et al. (2008) [13]	350 cases; 350 controls	Pregnant women delivered live births, Negeri Sembilan; mean age 28 years; Malay (63%), Chinese (12%), Indian (20%), others (5%)	Case-control study	2 = moderate; Study design = 1, sample size =1	Retrospectively retrieved from medical records	- Pre-pregnancy weight (kg) based on measured weight in the first trimester or recalled weight at late booking; - blood pressure	Birth	LBW < 2.5 kg vs. birth weight ≥ 2.5 kg	Higher pre-pregnancy weight was associated with lower odds of LBW.
Rahman et al. (2008) [14]	312 cases; 312 controls	Healthy pregnant women with singleton live births, Kedah; age < 20y (2%), 20-34y (80%), ≥35y (18%); Malay (75%), Chinese (10%), Indian (14%), others (1%)	Case-control study	2 = moderate; Study design = 1, sample size =1	Retrospectively retrieved from medical records	- GWG (< 10, 10–12, > 12 kg) based on difference between measured weight at last antenatal visit and recalled pre-pregnancy weight; - blood pressure	Birth	LBW < 2.5 kg vs. birth weight 2.5–4.0 kg	Low GWG was associated with higher odds of LBW.
Tan et al. (2009) [15]	1368	Pregnant women with singleton births, Kuala Lumpur; mean age 30y; Malay (62%), Chinese (18%), Indian (15%), others (5%)	Cross-sectional study	2 = moderate; Study design = 1, sample size =1	Retrospectively retrieved from medical records	- GCT positive if 1-h plasma glucose ≥7.2 mmol/l.; - GCT false-positive (GCT positive & GTT negative); GDM (GCT & GTT positive), diagnosed based on WHO 1999	Birth	Birth weight; LBW (< 2.5 kg); macrosomia (> 4.0 kg);	GCT false-positive status was associated with higher odds of PTB. GDM was associated with higher odds of macrosomia babies, labour induction and caesarean section.
Rozlan et al. (2012) [16]	436	Singleton pregnancy women, 4 BMI groups; Underweight (< 18.5 kg/m^2^), Normal (18.5–24.9 kg/m^2^), Overweight (25–30 kg/m^2^), Obese (30 kg/m^2^) *according to WHO	Retrospective study	1 = poor; Study design =1	Pre-pregnancy until prior to birth	- Maternal data of height and weight gain obtained by phone called interview.	Birth	Birth weight	Normal BMI with low GWG was associated with higher odds of PTB and LBW.
Loy et al. (2013) [17]	121	Malay healthy pregnant women with singleton full-term births, Kelantan; age 19–40 years	Cross-sectional study	2 = moderate; Study design = 1, valid data collection method =1	Throughout pregnancy	Energy and nutrient intake (unit/day); fruit and vegetable intake (g/day) based on semi-quantitative food frequency questionnaire	Birth	Birth weight	Greater leafy vegetable intake was associated with larger head circumference (HC). Greater tuber vegetable intake was associated with higher birth length and larger HC. Greater fruit intake was associated with higher birth weight, birth length and HC.
Kampan et al. (2013) [18]	400 cases; 400 controls	Pregnant women with diabetes and healthy control, Kuala Lumpur; age < 25y (8%), 25-35y (73%), >35y (19%),; Malay (69%), Chinese (25%), Indian (5%), − others (1%)	Case-control study	2 = moderate;; Study design = 1, sample size =1	Retrospectively retrieved from medical records	Diabetes in pregnancy (Type 1/ 2 diabetes) and GDM (insulin; diet control). Diabetes in pregnancy diagnosis based on fasting glucose ≥6.0 mmol/l and 2-h post-load 75 g glucose ≥7.8 mmol/l. GDM diagnosis criteria not specified.	Birth	IUGR; macrosomia (> 4.0 kg);	Women with diabetes on insulin had higher odds PTB. Higher rates of macrosomia, low Apgar score, NICU admission, hypoglycaemia and RSD were shown in women with diabetes, especially in those on insulin. Higher rates of NICU admission, macrosomia and RDS were shown in women with higher HbA1c.
Ismail et al. (2013) [19]	279	Pregnant women at risk of diabetes with singleton births, Kuala Lumpur; mean age 31.1y; Malay (76%), Chinese (21%) Indian (3%)	Prospective cohort study	2 = moderate; Study design = 1, valid data collection method =1	Not specified	GDM diagnosis based on fasting glucose ≥6 mmol/l or 2 h post-load 75 g glucose ≥7.8 mmol/l; maternal fasting serum insulin and HOMA-IR score	Birth	Macrosomia (> 4.0 kg)	High HOMA-IR score (≥2.92 vs. < 2.92) was associated with higher rates of neonatal hypoglycaemia and caesarean section in GDM women.
Yadav and Lee (2013) [20]	666	Healthy pregnant women with singleton live births, Negeri Sembilan; age < 24y (18%), 25-29y (41%), 30-34y (24.8%), ≥35y (17.0); Malay (68%), Chinese (14%), Indian (14%)	Cross-sectional study	2 = moderate; Study design = 1, sample size =1	Retrospectively retrieved from medical records	Pre-pregnancy BMI (< 20, 20–24.9, ≥25 kg/m^2^) based on first booking measured weight and height; blood pressure	Birth	LBW < 2.5 kg	Low pre-pregnancy BMI (< 20 vs. 20–24.9 kg/m^2^) was associated with higher odds of LBW.
Manaf et.al (2014) [21]	236	Pregnant women, Underweight (12.7%) Normal (55.1%) Overweight (25%) Obese (7.2%)	Cross Sectional	2 = moderate; Study design = 1, sample size =1	Throughout pregnancy	Nutritional knowledge and dietary intake	Prior to Birth	Micronutrient intake	One-third of respondents were anaemic. Most of the participants did not achieve RNI for calcium, folic acid, niacin and vitamin D.
Jan Mohamed et.al (2014) [22]	102	Pregnant women aged 19–40 years.	Prospective cohort study	2 = moderate; Study design = 1, sample size =1	14–24 gw; ≥32 gw	Blood and breast milk vitamin D levels; Dietary intake	14–24 weeks; ≥32 gw	Dietary calcium intake	Vitamin D deficiency [25(OH) D < 50 nmol/L] was detected in 60 and 37% of women in the second and third trimesters of pregnancy, respectively. Higher maternal serum vitamin D level in the second trimester of pregnancy was associated with an elevated level of vitamin D in breast milk at delivery (β = 0.002, *p* = 0.026).
Yadav and Lee (2014) [23]	2332	Pregnant women with singleton full-term births, Negeri Sembilan; Malay (67%), Chinese (14%), Indian (15%), others (4%)	Cross-sectional study	3 = high; study design = 1; sample size =1; valid data instrument = 1	Retrospectively retrieved from medical records	Pre-pregnancy BMI (< 20, 20–24.9, ≥25 kg/m^2^) based on first booking measured weight and height, GWG (< 10, ≥10 kg), diabetes in pregnancy	Birth	Macrosomia > 4.0 kg	High pre-pregnancy BMI (≥25 vs. < 20 kg/m^2^) was associated with higher odds of macrosomic babies. High GWG (≥10 vs. < 10 kg) was associated with higher odds of macrosomic babies. Diabetes in pregnancy was associated with higher odds of macrosomic babies.
Yeop et.al (2018) [24]	396	Pregnant women aged 18–40 years	Cross-sectional study	3 = high; study design = 1; sample size =1; valid data instrument = 1	First trimester.	Milk intake and prevalence of hypocalcaemia on serum calcium	First trimester pregnancy	Calcium intake	Hypocalcemia was present in 26% of pregnant women. Median calcium intake in food was 625.53 mg/day. Pregnant women who consumed less than two glasses of milk per day had two times higher risk of developing hypocalcaemia.
Kaur et al. (2019) [25]	437	Pregnant women at ≥20 gw in urban and rural	Prospective cohort study	3 = high; study design = 1; sample size =1; valid data instrument = 1	During pregnancy and followed up after they had given birth.	Questionnaires on sociodemographic characteristics and physical activity. Weight and middle-upper arm circumference were measured.	Birth	LBW	Rural women had more LBW infants than urban women. Rural women were less sedentary and participated in more household/caregiving activities, sports activities and less occupational activity than urban women. Older age, low parity and low MUAC increased the risk of LBW infants in rural, but not in urban women
Edi (2021) [26]	483	Pregnant women with singleton full-term births, Kuala Lumpur and Selangor; ≥ 18 years, ≥ 28 weeks of gestations.	Cross-sectional study	3 = high; study design = 1; sample size =1; valid data instrument = 1	During pregnancy and follow up after they had given birth.	Pre-pregnancy BMI obtained from maternal medical records; Total gestational weight gain; prenatal care visit history; maternal smoking and SHS exposure during pregnancy	Birth	Birth weight	Inadequate GWG and exposure to SHS at home were significant predictors of LBW.
Hasneezah et al. (2020) [27]	162	Anaemic pregnant women with haemoglobin (Hb) level between 7. 0 g/dl and 11.0 g/dl, singleton pregnancy; Malay (72.8%), Chinese (5.6%), Indian (16.0%) and others (4.9%).	Quasi-experimental study	3 = high; study design = 1; sample size =1; valid data instrument = 1	Second and third trimester	Haemoglobin level, knowledge score, Health Belief Model construct score, dietary intake	35–37 weeks	Dietary iron intake	The dietary iron intake of respondents at pre- and post-test was below the Malaysian recommended iron intake for pregnant women. Low intake of iron could be due to the lower protein intake limited by economic status
Yong et al. (2020) [28]	2193	Healthy, non-diabetic women, singleton pregnancy; Malay (83.7%), Chinese (4.7%), Indian and others (11.6%).	Retrospective cohort study	2 = moderate; study design =1; sample size = 1	Throughout pregnancy	Rate of gestational weight gain and total gestational weight gain	Birth	Birth weight	Higher GWG as well as increasing GWG trajectories was associated with higher risk of PTB and LBW. Women with maintained rate of GWG at an average of 0·58 kg/week had lower risk of having SGA infants.
Woon et al. 2019 [29]	535	Pregnant women with singleton pregnancy, ≥ 18 years, ≥ 28 weeks of gestation.	Prospective cohort study	3 = high; study design = 1; sample size =1; valid data instrument = 1	Third trimester	Serum 25(OH) D level, maternal vitamin D intake and supplementation, and sun exposure.	Prior to Birth	Vitamin D intake	Three-quarters of respondents did not achieve the RNI for vitamin D. Food sources, namely fish and fish products accounted for major source of vitamin D.

*Abbreviations*: *BMI* Body mass index, *FFQ* Food frequency questionnaire, *GCT* Glucose challenge test, *GDM* Gestational diabetes mellitus, *GTT* Glucose tolerance test; gw, gestation week, *GWG* Gestational weight gain, *HOMA-IR* Homeostatic model assessment of insulin resistance, *IUGR* Intrauterine growth retardation, *LBW* Low birth weight, *MUAC* Mid-upper arm circumference, *WHO* World Health Organization

### Adequacy of dietary intake

Table 3 shows the results of studies that examined micronutrient intakes among pregnant women. Only 5 studies were identified. Studies consistently showed that pregnant women did not meet the Recommended Nutrient Intakes (RNI) for dietary calcium [21, 22, 24], vitamin D [21, 24, 29], and iron [21, 27].

### Nutritional factors and infant birth weight

The results of studies that examined the association of nutritional factors (maternal anthropometry and food group intake) with infant birth weight were included in Table 3. Fourteen studies were identified. Studies showed an association between high maternal BMI and macrosomic babies, while low maternal BMI was associated with LBW babies.

### Nutritional factors and infant birth weight

The studies that examined the association of nutritional factors (maternal anthropometry and food group intake) with infant birth weight were included in Table 3. Twelve studies were identified; five of them investigated pre-pregnancy BMI and GWG, four studied gestational diabetes mellitus (GDM), and one examined hypertension among pregnant women. However, there was only one study that examined MUAC and food group intake. Studies showed an association between high maternal BMI and macrosomic babies, while low maternal BMI was associated with LBW babies.

### Maternal anthropometry and birth weight

#### Pre-pregnancy BMI

High pre-pregnancy body weight and BMI were associated with less LBW deliveries [13, 20, 30], but increased the birth of macrosomic babies [23]; while low pre-pregnancy BMI increased LBW deliveries [20].

#### GWG

Excessive GWG was associated with increased frequency of macrosomia [23], particularly evident in overweight and obese pregnant women [16] while insufficient GWG was associated with increased frequency of LBW babies, independent of pre-pregnancy BMI [16].

#### Mid-upper arm circumference (MUAC)

One study showed that low MUAC was associated with increased LBW deliveries in rural, but not in urban women [25].

### Food group intake and birth weight

One study showed that higher fruit intake was associated with greater birth weight, while intakes of confectioneries and condiments were both associated with lower birth weight [17].

### Nutrition-related co-morbidities in pregnancy and infant birth weight

The results of studies that examined the effects of co-morbidities (hyperglycemia and hypertension during pregnancy) on infant birth weight were shown in Table 3. Maternal hyperglycemia, defined as gestational diabetes mellitus (GDM) or high plasma glucose, was associated with increased macrosomic or large-for-gestational-age deliveries in three [15, 18, 23] out of four studies. There was no association between maternal hyperglycemia and LBW [13, 15, 18]. Maternal hypertension, defined as high systolic and/ or diastolic blood pressure, was associated with increased LBW deliveries in three studies [13, 14, 20].

### Quality appraisal and study findings

Out of the 17 studies, 10 (59%) scored 3, meaning high quality and 6 (35%) scored 2, meaning moderate quality, and only one study [16] scored 1 or low.

The research findings were quite consistent with the quality of the studies. A couple of studies that were rated moderate [13, 20] and one, which was rated high [26] showed significant association of pre-pregnancy weight of women with low birth weight babies. Conversely, more vegetable and fruit intakes during pregnancy were associated with higher birth weight, higher birth length and higher head circumference in a study that was moderate in rating [17].

Two studies with good quality and four studies with moderate quality showed that factors associated with increased infant’s birth weight (macrosomia) were high pre-pregnancy BMI, excess GWG and high blood glucose levels.

Maternal hyperglycemia, defined as gestational diabetes mellitus (GDM) or high plasma glucose, was associated with increased macrosomic or large-for-gestational-age deliveries in three studies [15, 18, 23]. Women with diabetes, especially those on insulin were more likely to give birth to babies with macrosomia in two of the three studies rated moderate [15, 18]. In another study, rated high quality [23], high pre-pregnancy BMI (≥25 vs. < 20 kg/m^2^) was associated with higher odds of macrosomic babies.

Dietary iron intake was below the Malaysian recommended iron for pregnant women in a high quality study [27]. Similarly, low intake of vitamin D was identified in three-quarters of pregnant women compared with the recommended national intakes in another high quality study [29].

## Discussion

This review showed that maternal micronutrient intakes were below the recommended levels, specifically vitamin D, calcium and iron, as shown by two high quality studies [27, 29]. Two moderately rated studies and one high quality study reported that factors associated with increased infant’s birth weight (macrosomia) were high pre-pregnancy BMI, excess GWG and high blood glucose levels. Increased fruit intake also increased birth size among study samples comprised mainly of normal weight infants, which is only reported in moderate quality study [18]. Meanwhile, LBW was associated with low pre-pregnancy BMI, inadequate GWG, intake of confectioneries and condiments, and high blood pressure. These findings are contributed by four moderate quality studies.

In general, the findings of this review from Malaysia are in line with studies from both Asian and Western settings that report an unfavorable nutritional-related maternal environment during pregnancy including underweight or overweight/obesity, suboptimal GWG, poor dietary intake, hyperglycemia and/or hypertension were associated with abnormal birth weight deliveries [2–6, 31–35].

These results are consistent with the emerging evidence showing the associations of pre-pregnancy BMI, GWG, pre-existing diabetes, GDM and maternal dietary factors with adverse birth outcomes including LBW, preterm birth, macrosomia, adiposity, neonatal hypoglycemia and caesarean delivery.

### Dietary intake of pregnant women in terms of micronutrients

Hamid and colleagues reported earlier that among rural pregnant women, 37% displayed serum vitamin D deficiency in their third trimester, potentially resulting in low vitamin D concentrations in breast milk at birth [22]. Meanwhile, among urban pregnant women, hypocalcemia was shown in 26% women who were mostly deficient in vitamin D levels [23]. Both vitamin D and calcium complement each other and are important in maintaining bone health and reducing preeclampsia risk during pregnancy. It has been demonstrated that pregnant women consuming less than two glasses of milk per day were at higher risk of developing hypocalcemia [23]. Higher intake of dietary vitamin D has been shown to be associated with a lower odds of vitamin D deficiency during pregnancy [29]. Emphasizing greater intake of vitamin D through diet could help as Malaysian women have been studied to have low sunlight exposure resulting in higher vitamin D deficiency [36]. Hence, efforts to supplement or improve dietary sources of vitamin D are needed as vitamin D has been established to help reduce pregnancy complications [24].

Multiple reviews have consistently revealed that suboptimal intakes of vitamin D, calcium and iron, are widely prevalent among pregnant women especially in those from low- and middle-income countries [37–39]. More studies are warranted to explore the role of sunlight exposure and dietary vitamin D intake towards vitamin D status among pregnant women.

The majority of pregnant women in Malaysia were not able to achieve their recommended intake for iron through the diet [21, 27, 40], which is the same scenario as observed in other countries [41, 42]. This is particularly evident in Indian ethnicity, exposing Indian women to a higher risk of developing iron-deficiency anemia during pregnancy [43–45]. This could be due to the common practices of vegetarian diet among Indians who do not receive adequate iron-rich foods in their meals [46]. Poverty, low education, and lack of iron-rich sources in food intake are often regarded as reasons to poor dietary intake of iron among women in developing nations [41, 45, 47, 48]. Thus, education on iron-rich food intake and iron supplementation compliance should be emphasized for improved maternal iron status [49]. Previous study demonstrated the effectiveness of Health Belief Model-based educational intervention in improving the compliance to iron supplementation among pregnant women, highlighting the potential of reducing anemia through encouraging preventive healthy behaviours [27]. Currently, there is a lack of studies focusing on obesity with anemia among pregnant women although obesity is an important risk factor for anemia [50]. As such, with the rising trend of obesity in Malaysia [51], future research should focus on studying the role of obesity in anemia development among pregnant women.

### Nutritional factors and their association with offspring birth weight

#### Anthropometry

The few studies have shown that maternal pre-pregnancy BMI and GWG are positively associated with birth weight among Malaysian women. An evidence derived from the only prospective cohort study in this review [30] showed that infants born to mothers of higher pre-pregnancy BMI were consistently heavier throughout their first year of life. This is consistent with pooled data from both developed and developing countries, reporting a 1.5-fold higher risk of LBW in underweight women compared with those of normal weight [52]. Besides, earlier studies found that those women with low GWG (< 10 kg) and high GWG (≥10 kg) were more likely to have LBW and macrosomia, respectively [14, 23]. A later study using updated Institute of Medicine (IOM) guidelines in 2009 showed that only among women with normal pre-pregnancy BMI (18.5–24.9 kg/m2), low GWG (< 11.5 kg) was associated with LBW, as well as PTB [16]. A recent study also supported the association between inadequate GWG and LBW [26]. An evidence from meta-analysis indicated that women with GWG above and below IOM guidelines had approximately 2-fold higher risk of delivering macrosomic and small-for-gestational age/PTB babies, respectively [53]. Irregular and higher GWG trajectories throughout the second and third trimesters were associated with LBW and PTB, respectively [28]. Hence, measuring the GWG trajectory may be more useful to provide information on timely weight management among pregnant women.

#### Food group intake

Individual nutrients or foods were examined in two studies in Malaysia, whereby more fruit and vegetable intake at late pregnancy were associated with larger birth sizes [17, 54]. Though single food item was studied instead of the whole diet, these studies were supported by another study reporting that maternal dietary pattern at late-second trimester with high intake of vegetables, fruit, white rice, and low intake of fast foods and flavored rice were associated with large birth size and lower PTB risk [31]. Similarly, as reviewed by another study, improved diet quality during pregnancy was associated with longer and heavier babies but within normal growth [55]. The protective benefits of fruits and vegetables against poor birth outcome should be further explored particularly in understanding the mechanistic biological effects.

### Nutrition-related co-morbidities during pregnancy and their association with offspring birth weight

#### High blood glucose

Malaysian pregnant women with diabetes, including both pre-existing diabetes and GDM were associated with macrosomia [15, 18]. Promising evidence has been emerged from the multicenter study [56] and systematic review worldwide [32], showing there is a consistent graded linear association between maternal glucose levels during pregnancy and birth weight/adiposity. However, such a continuous effect of hyperglycemia on adverse birth outcomes have not been investigated across Malaysian studies. Neonatal hypoglycemia, PTB, cesarean section and induction of labor are also the risks for women with diabetes, as reported here [15, 18, 19] and other review [32]. This reflects that overall outcomes of women with diabetes remained poor though under dietary and/ or insulin management. In addition, study by Muna et al. [57] observed that there was a reduction in serum leptin levels among GDM women on a controlled diet. Generally, women with GDM had higher circulating leptin in comparison to normal pregnant women, emphasizing the importance of dietary management in controlling metabolic profile [58].

A study by Ismail and co-researchers suggests that higher BMI status in women with diabetes predisposes them to a higher insulin resistance state [19]. Additionally, higher rate of obesity (~ 50%) was observed in women with diabetes compared with healthy control [18, 19]. Overweight/ obese women with diabetes tended to have a higher insulin resistance during pregnancy than those healthy counterparts, associating it with pregnancy complications [19]. This suggests the importance of having optimal maternal weight to reduce the risk of pregnancy complications.

#### High blood pressure

Hypertension in pregnancy includes gestational hypertension, preeclampsia and eclampsia, were associated with LBW deliveries among Malaysian women. This is in agreement with previous reports from both developed and developing countries, showing high rate of LBW or small-for-gestational-age in women developing hypertension in pregnancy [33–35]. Not only imposing adverse effect on fetal growth, but hypertension in pregnancy has also been shown to influence long-term cardio-metabolic health outcomes in the offspring. This is evidenced by the consistent association shown between gestational hypertension and a higher offspring blood pressure as documented by a recent systematic review [59]. Indeed, a population-based cohort in Sweden (*n* = 13,893) further demonstrated that adult offspring of mothers with hypertension in pregnancy had an adverse cardio-metabolic trajectory, including higher risk of hypertension, higher BMI, higher plasma glucose at age of 40 years, compared with offspring of mothers who did not have hypertension in pregnancy [60]. These associations may be potentially mediated by the intrauterine growth restriction as indicated by LBW which warrant further investigations.

Only a limited number of research related to maternal diet and nutrition were identified over the past decades. Most of the studies in this review focused on iron-deficiency anemia and vitamin D deficiency. There is an urgent need to investigate the role of other dietary patterns and micronutrient deficiencies in determining birth outcomes among Malaysian women. Furthermore, the focus of Malaysian studies was more on certain maternal nutrition-related indicators rather than other aspects of maternal nutrition status, nutrient intake, and dietary pattern. Dietary factors influence towards maternal nutritional status remains understudied in Malaysia with most studies being cross-sectional instead of longitudinal. More attention should be focused on a wider range of micronutrient deficiencies. Importantly, more research is required, particularly prospective cohort study, case-control study, and randomized control trial of maternal nutritional intervention, to explore the maternal dietary practices and to understand the effects of maternal nutritional status on birth outcomes. This is critical in developing targeted interventions for Malaysian women of reproductive age, in order to initiate appropriate health management as early as possible to improve offspring outcomes.

### Limitations

Despite the information compiled in this review involves a total of 11,870 sample population, several limitations including the study quality are subjected to bias. The scope of this review was limited to the Malaysian population. The study results must not be generalized to other populations. Secondly, because of the nature of a scoping review, multiple structured searches are required. Even though this review identified articles using 10 or more sources, there is always a possibility that some of the important articles could have been missed. The absence of current studies is a limitation. Another limitation could be the lack of training of the reviewers on quality appraisal prior to the research. We suggest such training of the reviewers should reduce the risk of bias and ensure reliability and consistency in the quality appraisal ratings. The scale that was used to assess risk of bias could be rather insensitive – there were only four items, and the type of the study design (e.g. cross-sectional, case-control and cohort studies) were all weighted equally.

Although the findings of maternal nutritional-related indicators such as pre-pregnancy BMI, GWG, glucose and dietary intake in relation to birth outcomes that were covered under this review have been supported by literature, the comparability between the included studies is constrained by a limited number of available reports, along with variations in study design, variable assessment method and period across studies. Dietary factors influence towards maternal nutritional status remains understudied in Malaysia with most studies being cross-sectional instead of longitudinal. More attention should be focused on a wider range of micronutrient deficiencies. Importantly, more research is required, particularly prospective cohort study, case-control study and randomized control trial of maternal nutritional intervention, to explore the maternal dietary practices and to understand the effects of maternal nutritional status on birth outcomes. This is critical in developing targeted interventions for Malaysian women of reproductive age, in order to initiate appropriate health management as early as possible to improve offspring outcomes.

## Conclusions and recommendations

This review demonstrated that maternal nutrition status plays a significant role in birth outcomes related to infant birth weight. Reviewed studies consistently showed that increased infant birth weight (macrosomia) was associated with high pre-pregnancy BMI, excess GWG and high blood glucose levels. At the same time, a few studies also demonstrated the association of poor maternal nutritional status (such as low MUAC) with adverse pregnancy outcome of LBW.

This review reaffirms the importance of maternal nutrition on maternal and infant outcome but highlights that this area remains understudied in Malaysia. Therefore, there is a need to explore other aspects of maternal nutrition before and during pregnancy. Findings of this review could support the planning or future research and interventions for Malaysian population, both in the rural and urban setting. Meanwhile, the role of dietary factors on maternal health outcomes including maternal nutritional, metabolic, mental and overall wellbeing should be emphasized. This is critical in developing targeted interventions for Malaysian women of reproductive age, in order to initiate appropriate health management as early as possible to improve the outcomes of the offspring.

Although the neighboring countries such as Thailand and Indonesia have almost a similar prevalence of LBW as compared with Malaysia, further studies may be undertaken to compare the determinants associated with LBW in the three neighboring countries.

## Data Availability

Data sharing is not applicable to this article as no datasets were generated or analysed during the current study.
